# Brain volumes and cortical thickness and associations with cognition in children born extremely preterm

**DOI:** 10.1038/s41390-024-03480-1

**Published:** 2024-08-21

**Authors:** Hedvig Kvanta, Nelly Padilla, Daniela Nosko, Gustaf Mårtensson, Lina Broström, Lexuri Fernández de Gamarra-Oca, Jenny Bolk, Ulrika Ådén

**Affiliations:** 1 Department of Women’s and Children’s Health, Karolinska Institute, Solna, Stockholm Sweden; 2 Paediatric Department, Örebro University Hospital, Örebro, Sweden; 3 Department of Psychology, Faculty of Health Sciences, University of Deusto, Bilbao, Bizkaia Spain; 4 Clinical Epidemiology Division, Department of Medicine, Karolinska Institute, Stockholm, Sweden; 5 Department of Clinical Science and Education, Södersjukhuset, Stockholm, Sweden

## Abstract

**Background:**

Children born extremely preterm (EPT) have altered brain volumes and cortical thickness and lower cognition than children born at term. Associations between these have remained largely unexplored, due to the lack of studies focusing on children born EPT.

**Methods:**

Children underwent brain magnetic resonance imaging (MRI) at term and/or 10 years and cognitive assessments at 12 years. The study comprised of 42 children born EPT and 29 term-born controls with cognitive data and MRI data at 10 years, 25 children born EPT had MRI data at term age and 20 had longitudinal MRI data.

**Results:**

Cognition was positively associated with brain volumes at 10 years, but negatively associated with cortical thickness at 10 years. Most associations between term age brain volumes and cognitive outcomes were non-significant for children born EPT. Growth from term to 10 years in children born EPT was not associated with cognition. Insular volume was positively associated with cognition in children born EPT.

**Conclusion:**

Imaging assessments at 10 years had similar associations to cognition in children born EPT and term-born controls. Insular volume could be a biomarker for cognitive outcome. Associations between brain volumetric growth and cognition require further investigation.

**Impact:**

- This study investigated brain volumes, volumetric growth, and cortical thickness in children born extremely preterm, who have rarely been studied exclusively, and compared the data with term-born controls.
- In both groups, brain volumes at 10 years were positively associated with cognitive outcome at 12 years, but cortical thickness at 10 years was negatively associated with cognitive outcome at 12 years. Volumetric growth from term age to 10 years was not associated with cognitive outcome in the subset of children born extremely preterm with longitudinal data.
- Insular volume may be a potential biomarker for cognitive outcome in children born extremely preterm.

## Introduction

Children born preterm are exposed to an early *ex-utero* environment and need advanced life-support. They experience altered sensory stimuli and have a high risk of comorbidities associated with prematurity.^1^ The second half of pregnancy is a critical period for brain development, characterized by axonal growth, neural migration and differentiation, synaptogenesis, and subplate growth.^1^ Depending on their gestational age (GA) at birth, children born extremely preterm (EPT) may spend three, or even four, months during this important period outside the womb. Children born with EPT have an increased risk of an impaired neurodevelopmental outcome.^2^

It has been reported that children born very preterm and EPT, at less than 32 and 28 weeks, respectively, had altered brain volumes at term age and later childhood than those born at term.^3–6^ Most studies have reported that time did not compensate for the total brain tissue reduction at term age.^4,6^

Evidence to date has indicated that total brain tissue volumes at term age were associated with cognitive outcomes in early childhood in children born very preterm.^3,4^ There has been less research on children born EPT, but our group has reported that brain volumes at term age were associated with neurodevelopmental outcomes at two years of age.^7^ Few studies have explored the relationships between the brain volumes of children born EPT, when they reached term age, and cognitive assessments beyond early childhood.^3^

Total brain tissue volumes in childhood and adolescence of grey matter (GM) and white matter (WM) for very preterm children have also been associated with cognitive outcomes,^4,5,8^ but only one study exclusively examined total brain volumes in children born EPT.^9^ The authors reported a positive association between total brain tissue volumes and full-scale intelligence quotient (FSIQ). However, the cohort was born in the early 1990s, and medical care for children born EPT has drastically changed since then.^9^

We know from previous studies that brain volumes for children born very preterm and EPT are affected with regional variability.^8,10^ Voxel-based morphometry (VBM) is a method that enables voxel-wise comparisons of brain tissue volumes, and is valuable in regional brain volume analyses.^11^ There has been a lack of modern cohort studies that have investigated the associations between total and regional volumes in childhood and cognition in children born EPT.

There are longitudinal studies that explored volumetric growth in very preterm children, from term age to childhood, and how it was related to cognitive outcomes. One study reported that there were no significant associations between the growth of GM or WM and cognitive assessments at seven years of age.^4^ Another study reported that regional volumetric growth patterns from seven to 13 years showed different associations with cognitive outcomes in very preterm and term-born children.^12^

Cortical thickness is the distance between the GM and WM border and the border between GM and the pial surface.^13^ Global cortical thickness increases during infancy and the first years of life and then the growth rate declines from early childhood, as cortical thinning becomes prominent.^13,14^ Cortical thinning reflects the pruning of brain connections and myelination and is considered an important developmental step.^14^ There has been limited research on the relationships between cortical thickness and cognition in children born EPT. There have been reports of positive associations between cortical thickness and cognition in children born with a very-low-birth-weight.^15^ On the other hand, studies have also reported negative associations between cortical thickness and cognition in children born very preterm and at term.^16,17^

The main aim of this study was to examine the associations between MRI assessments at around 10 years (total GM and WM brain volumes, regional brain volumes, and mean cortical thickness) and cognition at 12 years of age in children born EPT and compare these with term-born controls.

As secondary objectives we sought to relate total brain volumes of GM and WM measured in children born EPT at term age, to cognition at 12 years of age. We also aimed to study how brain volume growth from term age to around 10 years of age was related to cognition in children born EPT.

## Materials and methods

### Study population and study design

This was a prospective, observational, population-based cohort study of children born in Stockholm, Sweden, before 27 weeks and 0 days between January 1, 2004 and March 31, 2007.

There were 128 children born EPT during the study period who were alive at term age, defined as 40 weeks and 0 days. We asked the parents for permission for the children born EPT to undergo MRI scans at term age and 10 years of age and developmental assessments at 12 years of age.

Children with severe medical conditions were excluded. These were defined as chromosomal abnormalities, congenital malformations, periventricular leukomalacia on cranial ultrasound, intraventricular hemorrhage (IVH) grade III, and periventricular hemorrhagic infarctions diagnosed with ultrasound in the neonatal period or marked ventricular dilatation. We also excluded children with focal brain lesions and severe WM abnormalities, according to a previously defined scoring system.^18^ All the images were visually inspected and those that failed to meet the quality criteria, due to motion artifacts or blurring in the GM and WM interface, were excluded.

Drop-out analyses were performed for children who were included or not included due to either declined participation or with MRI images of too low quality to analyze.

The controls were singleton, term-born, healthy children who were identified from the Swedish Medical Birth Registry at 2.5 years of age. They were matched to the children born EPT by birth location, day of birth, maternal country of birth, and sex. This resulted in 77 Stockholm-born controls being invited to MRI scans at around 10 years of age, and cognitive assessments at 12 years. The controls had not undergone MRI scans at term age.

### Characteristics of study groups

Perinatal data were retrieved from medical charts. Sepsis was defined as a positive blood culture, or clinical symptoms combined with an elevated C-reactive protein or leukocyte count. Small for gestational age was a birthweight of less than 2 standard deviations below the mean.^19^ Necrotizing enterocolitis was defined using Bell criteria.^20^ Bronchopulmonary dysplasia was the need for oxygen at 36 weeks of gestation. Patent ductus arteriosus (PDA) was the need for PDA ligation or PDA treatment with ibuprofen. Maternal education was dichotomized as mothers that did or did not go to university.

### Brain MRIs at term age and at 10 years of age

At term age the three-dimensional T1 weighed images were acquired using a Philips Intera 1.5 MRI scanner (Philips International, Amsterdam, The Netherlands). The MRI protocol has previously been published.^6,21^

At 10 years of age the three-dimensional T1 weighted images were performed using a General Electric SIGMA HDx 3.0 Tesla system (GE Healthcare, Milwaukee, Wisconsin). The detailed protocol has been previously published.^6^

### Preprocessing of MRI images and calculating of brain volumes and cortical thickness

The preprocessing steps of the neonatal MRI scans have previously been published in detail and were performed by an experienced neuroscientist (NP).^21^ In brief, images were segmented into tissue classes, based on the guidance provided by a neonatal brain template.^21^ These segmented images were spatially normalized and modulated and brain volumes in cm^3^ were calculated for each tissue class. For the current study, we used total GM, obtained by adding cortical and subcortical GM, and total WM for the analyses.

The preprocessing steps for calculating the brain volumes at 10 years of age has previously been described.^6^ Briefly, the pipeline involved reorientation, brain extraction, spatial normalization and segmentation using guidance from age-specific tissue priors and spatial normalization using the DARTEL algorithm.^22^ The images were then modulated and brain volumes in cm^3^ were calculated for tissue classes. For brain volume growth calculation, the term age volumes were subtracted from the volumes at 10 years of age for GM and WM respectively.

Cortical reconstruction and cortical thickness calculations were performed with FreeSurfer version 7.2.0 (Harvard University, Cambridge, Massachusetts). The technical details of the cortical reconstruction and cortical thickness estimates have previously been described in detail.^23,24^ This study used the mean cortical thickness for each hemisphere.

### Cognitive assessment

Both groups were assessed with the Wechsler Intelligence Scale for Children, Fifth Edition (WISC-V) at 12 years of age.^25^ This measures cognitive ability by using five indexes: verbal comprehension, visual-spatial index, fluid reasoning index, working memory and processing speed. These indexes are used to calculate the FSIQ, with a mean of 100 and SD of 15. All the cognitive assessments were conducted by a certified psychologist.

### Statistics

All statistical analyses were performed using SPSS version 28 (IBM Corp, New York). Group comparisons of the characteristics between children born EPT and term-born controls and drop-out analyses were performed. The Student’s *t*-test or Mann-Whitney *U* test was used for the continuous variables and Pearson’s chi-square test or Fisher’s exact test was used for categorical variables, as appropriate. The total brain volumes at term age and at 10 years have been previously published.^6,21^

The associations between brain measurements at 10 years of age and cognitive outcomes at 12 years were explored for the whole study population of children born EPT and the term-born controls. The measurements were GM volume, WM volume, right and left mean thickness and the cognitive outcomes were FSIQ and WISC-V index scores. We used multiple linear regression, fitted using generalized estimating equations (GEE) with robust sandwich standard errors, to allow for clustering of multiple births within a family.

The cognitive outcomes were the dependent variables, and the brain volume or cortical thickness were the independent variables. Analyses were adjusted for the EPT and control groups, sex, age at the time of scan, and maternal education. Analyses were repeated controlling for GA at birth. Interactions between the imaging measurements and group (EPT or control), and imaging measurements and sex, were explored in the model. They were considered significant if *p* < 0.05 and insignificant interactions were removed from the model. Explanatory variables were selected based on earlier research findings and clinical relevance.^7,14,26^ The likelihood-ratio chi-square test for the full model was used at the outset to determine if the overall model was significant.

The β value represents changes in the cognitive assessment score for one-step change in brain volume (cm^3^) or cortical thickness (0.1 mm), when the other variables were held constant.

All the data were graphically inspected using scatter plots and box plot diagrams. No obvious outliers were detected, and all the children were kept in the final analyses. One child born EPT had a FSIQ < 70. The analyses were repeated without this individual and, because the results did not change, this child was kept in the analyses.

We then used GEE to explore any associations between brain volumes at term age, and cognitive outcome at 12 years, for the 25 children born EPT who had MRI data at term. The cognitive assessment was the dependent variable and brain volumes were the independent variables. These analyses were adjusted for sex, maternal education, and GA at the time of the scan. The analyses were repeated controlling for GA at birth.

We also performed GEE analyses with cognitive outcome as the dependent variable and longitudinal brain volume growth from term age to 10 years of age as the independent variables for the 20 children born EPT with MRI data at both time points. These analyses were adjusted sex, maternal education, and the children’s ages at the time of the MRI. We repeated the analyses controlling for GA at birth.

Corrections for multiple comparisons were applied using the Benjamini-Hochberg procedure with a false discovery rate of 0.05.^27^ Given the exploratory nature of this study, the interpretation was based on the directions, patterns, and magnitude of the findings, rather than specific *p*-values.^28^

### Voxel-based morphometry analysis

The VBM toolbox for SPM 12 software (Wellcome Department, University College London, London, UK) was used for the correlations between regional GM and WM brain volumes at 10 years and FSIQ at 12 years of age. The images imported into the VBM toolbox were segmented, modulated, and smoothed with a 6 mm Gaussian kernel. We tested both negative and positive correlations with FSIQ. The added covariates were sex, age at MRI, and maternal education. The analyses were also repeated adding GA at birth as a covariate. We analyzed the EPT and term-born control groups separately. For statistical purposes, we used an initial voxel-level threshold at *p* < 0.001, the results were then corrected at cluster level using family wise error rate at *p* < 0.05, to account for multiple comparisons. The absolute threshold masking was set to 0.1 to avoid edge effects between the tissues. The Talairach atlas, registered to the Montreal Neurological Institute (MNI) space, and the Harvard-Oxford cortical and subcortical atlases were used for anatomical orientation.

## Results

### Study population

**Fig. 1:**
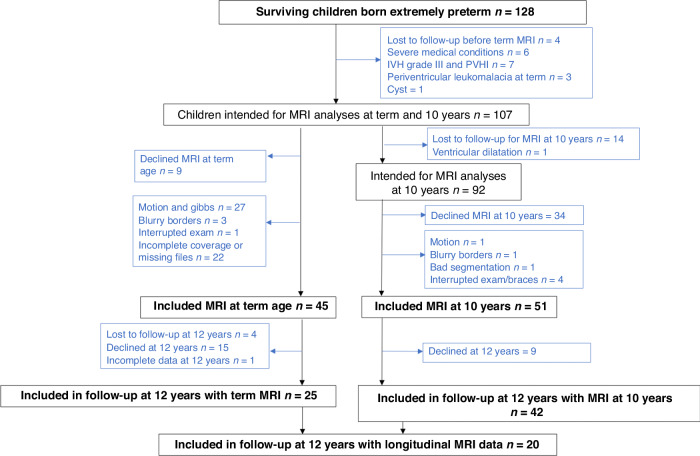
Flow chart of children born extremely preterm. Final inclusion of children born extremely preterm (EPT). 42 children had included MRI data at 10 years, 25 at term age, and 20 with MRI at both time points. All of these children had cognitive data at 12 years.

There were 128 children born EPT who survived and 45 of these had high-quality MRI data at term age and met the inclusion criteria for the study. They included 25 children who underwent cognitive assessments at 12 years of age (Fig. 1).

There were 51 children born EPT with high-quality MRI data at 10 years of age who met the inclusion criteria. The parents of nine of these children did not agree to them being followed-up at 12 years of age and 42 children born EPT had MRI data at 10 years and cognitive data.

There were 20 children born EPT who had longitudinal MRI data at term age and at 10 years of age and cognitive data at 12 years (Fig. 1).

There were 38 term-born controls with high-quality MRI scans at 10 years of age and 29 of these also underwent the cognitive tests at 12 years of age (Supplementary Fig. 1). None of these term-born controls had undergone MRI scans at term age.

The perinatal characteristics and drop-out analyses of the study groups are presented in Supplementary Tables 1–3. The drop-out analyses showed that the 20 children born EPT with longitudinal MRI data had a higher GA and a lower rate of bronchopulmonary dysplasia than the non-participants. There were no other significant differences.

### Group comparisons of characteristics, MRI data, and cognitive data

Table 1 compares data for the 42 children born EPT and the 29 term-born controls who had MRI scans at 10 years of age and cognitive data at 12 years of age. The EPT group had lower FSIQs than the term-born controls, with a mean difference of –14.2 points and 95% confidence interval (CI) of –20.8 to –7.5, *p* < 0.001. All five WISC-V index scores were significantly lower in the children born EPT than the term-born controls.

**Table 1:** Group comparisons of children born extremely preterm and term-born controls with MRI at 10 years and cognitive follow-up at 12 years.

	Children born EPT *n* = 42	Term-born controls *n* = 29	Mean difference, 95% CI	*p-*value^a^
**General characteristics**				
Age at scan in childhood, median (IQR)	10.5 (1.5)	10.3 (1.0)	–	^b^0.55
Age at 12-year-old follow-up, years, median (IQR)	12.2 (0.3)	12.1 (0.4)	–	^b^0.46
Gestational age, weeks, median (IQR)	25.6 (1.2)	40.0 (1.4)	–	^b^<0.001
Birth weight, grams, mean (SD)	839 (151)	3701 (432)	–	^a^<0.001
Male, *n* (%)	21 (50)	13 (45)	–	^c^0.67
Multiple births, *n* (%)	7 (17)	0	–	*–*
Maternal education, mothers attended university, *n* (%)	27 (64)	18 (62)	–	^c^0.85
**Cognitive assessment**				
FSIQ, mean (SD)	96.6 (14.8)	110.8 (12.3)	–14.2 (–20.8, –7.5)	^a^<0.001
Verbal comprehension index, mean (SD)	102.3 (16.9)	117.1 (15.9)	–14.9 (–22.8, –6.9)	^a^<0.001
Visual spatial index, mean (SD)	94.5 (15.8)	104.9 (12.6)	–10.4 (–17.4, –3.3)	^a^0.004
Fluid reasoning index mean (SD)	95.9 (12.1)	105.4 (10.6)	–9.5 (–15.0, –3.9)	^a^0.001
Working memory index, mean (SD)	91.3 (14.2)	99.3 (10.9)	–8.0 (–14.30, –1.8)	^a^0.013
Processing speed index, mean (SD)	94.5 (16.3)	105.9 (14.7)	–11.3 (–18.9, –3.8)	^a^0.004
**Imaging data**				
Grey matter, cm^3^, mean (SD)	745.9 (61.5)	771.9 (54.8)	–26.0 (–54.4, 2.4)	^a^0.072
White matter, cm^3^, mean (SD)	448.0 (42.0)	474.8 (32.0)	–26.8 (–45.2, –8.4)	^a^0.005
Intracranial volume, cm^3^ mean (SD)	1389.6 (118.0)	1453.0 (102.9)	–63.1 (–117.1, –9.2)	^a^0.023
Right hemisphere cortical thickness, mm, mean (SD)	2.78 (0.087)	2.83 (0.079)	–0.053 (–0.093, –0.012)	^a^0.011
Left hemisphere cortical thickness, mm, mean (SD)	2.78 (0.084)	2.84 (0.078)	–0.055 (–0.094, –0.015)	^a^0.007

^a^Student’s t-test, ^b^Mann-Whitney U test, ^c^Pearson Chi-Square

*FSIQ* full-scale intelligence quotient, *SD* standard deviation, *IQR* interquartile range, *EPT* extremely preterm.

The imaging parameters demonstrated that the children born EPT had significantly lower WM volume, intracranial volume (ICV) and bilateral mean cortical thickness than the term-born controls at 10 years of age.

### Brain volumes at 10 years of age and associations with cognitive outcomes at 12 years

**Fig. 2:**
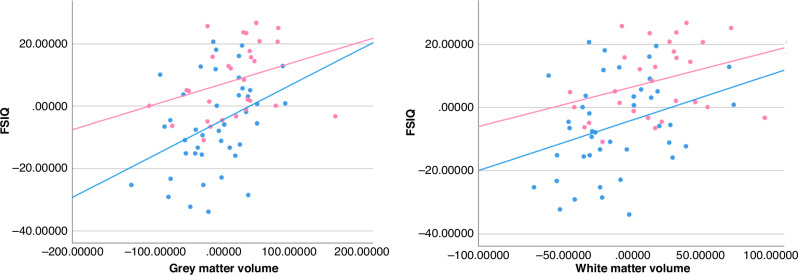
Scatter-plot of brain volumes at 10 years and FSIQ at 12 years for the 42 children born EPT and 29 term-born controls. The figure depicts associations between total grey matter (GM) and total white matter (WM) volumes at 10 years and FSIQ at 12 years for the children born EPT (blue) and term-born controls (pink). The unstandardized residuals were plotted after adjustments for sex, age at scan, and maternal education for brain volumes and after adjustments for sex and maternal education for FSIQ. The units on the y and x axes are FSIQ scores and cm^3^ respectively, both centered to have a mean of zero. FSIQ = full-scale intelligence quotient. EPT = extremely preterm.

The associations between brain volumes at 10 years and cognitive outcomes at 12 years are presented in Table 2. For the whole included study population of children born EPT and term-born controls the GM volume (β 0.091, 95% CI 0.031 to 0.15) and WM volume (β 0.13, 95% CI 0.045 to 0.22) were significantly associated with FSIQ after adjustments for group, sex, age at scan and maternal education (Table 2 and Fig. 2). The raw brain volumes for GM and WM volumes are plotted to the FSIQ in Supplementary Fig. 2. The GM and WM volumes were significantly positively associated with four of the five index scores (Table 2). All these associations remained significant after they were corrected for multiple comparisons. The results also remained after adjustments for GA at birth, Supplementary Table 4a).

**Table 2:** Associations between grey matter and white matter volumes at 10 years with cognitive outcomes at 12 years for the combined group of 42 children born extremely preterm and 29 term-born controls.

Children born EPT and term-born controls *n* = 71 (EPT *n* = 42 and term-born *n* = 29)						
	Grey matter			White matter		
	β	CI	*p-*value	β	CI	*p*-value
**FSIQ**	0.091	0.031; 0.15	0.003*	0.13	0.045; 0.22	0.003*
**Verbal comprehension**	0.070	–0.015; 0.15	0.11	0.069	–0.052; 0.18	0.28
**Visual spatial**	0.095	0.043; 0.15	<0.001*	0.12	0.041; 0.20	0.003*
**Fluid reasoning**	0.059	0.010; 0.11	0.018*	0.091	0.025; 0.16	0.007*
**Working memory**	0.070	0.026; 0.13	0.004*	0.12	0.040; 0.20	0.003*
**Processing speed**	0.074	0.011; 0.14	0.020*	0.14	0.035; 0.25	0.009*

The β value represents change in the cognitive assessment score for every cm^3^ increase in brain volume. Generalized estimating equations, dependent variable: cognitive outcome, independent variable: brain tissue in cm^3^; adjusted for group (EPT/control), sex, age at scan and maternal education. *= significant at *p* < 0.05, bolded values remained after correcting for multiple comparisons with the Benjamini-Hochberg procedure. *FSIQ* full-scale intelligence quotient, *EPT* extremely preterm.

Interactions between GM and WM brain volumes and group (EPT or term-born controls) or sex were added to the models, but none of the interactions were significant and were thus not kept in the final models.

### Cortical thickness at 10 years of age and associations with cognitive outcomes at 12 years

The associations between mean cortical thickness at 10 years of age and cognitive outcomes at 12 years are presented in Table 3. There were negative associations between the mean cortical thickness of the right hemisphere and FSIQ (β –4.3, 95% CI –8.7 to –0.015) and visual spatial index (β –4.9, 95% CI –9.1 to –0.67), after adjustments for the group, sex, age at the scan and maternal education. However, these associations did not remain significant after correcting for multiple comparisons. After adjustments for GA at birth the association between right hemisphere thickness and visual spatial index remained significant, while the associations with FSIQ remained in the same direction but did not reach statistical significance, Supplementary Table 4b).

**Table 3:** Associations between left or right hemisphere cortical thickness at 10 years with cognitive outcomes for 42 children born EPT and 29 term-born controls.

Children born EPT and term-born controls *n* = 71 (EPT *n* = 42 and term-born *n* = 29)						
	Mean cortical thickness, left			Mean cortical thickness, right		
	β	CI	*p-*value	β	CI	*p*-value
**FSIQ**	–2.9	–7.6; 1.7	0.22	–4.3	–8.7; –0.015	0.049*
**Verbal comprehension**	–2.4	–8.5; 3.5	0.43	–4.3	–10.1; 1.5	0.15
**Visual spatial**	–3.4	–7.8; 1.1	0.14	–4.9	–9.1; –0.67	0.023*
**Fluid reasoning**	–1.6	–4.7; 1.6	0.33	–2.4	–5.3; 0.49	0.10
**Working memory**	–1.3	–5.0; 2.5	0.50	–1.4	–4.7; 1.9	0.40
**Processing speed**	–0.98	–5.2; 3.2	0.65	–1.8	–5.9; 2.3	0.39

The β value represents change in the cognitive assessment score for every 0.1 mm increase in cortical thickness. Generalized estimating equations, dependent variable: cognitive outcome, independent variable: cortical thickness in 0.1 mm, adjusted for group (EPT/control), sex, age at scan and maternal education. *= significant at *p* < 0.05, bolded values remained after correction for multiple comparisons. FSIQ=full-scale intelligence quotient. *EPT* extremely preterm.

Interactions between mean cortical thickness and the EPT or control group, and the thickness and the child’s sex, were added to the models, but none of the interactions were significant and they were not kept in the final models.

### Regional brain volumes at 10 years of age and FSIQ at 12 years

**Fig. 3:**
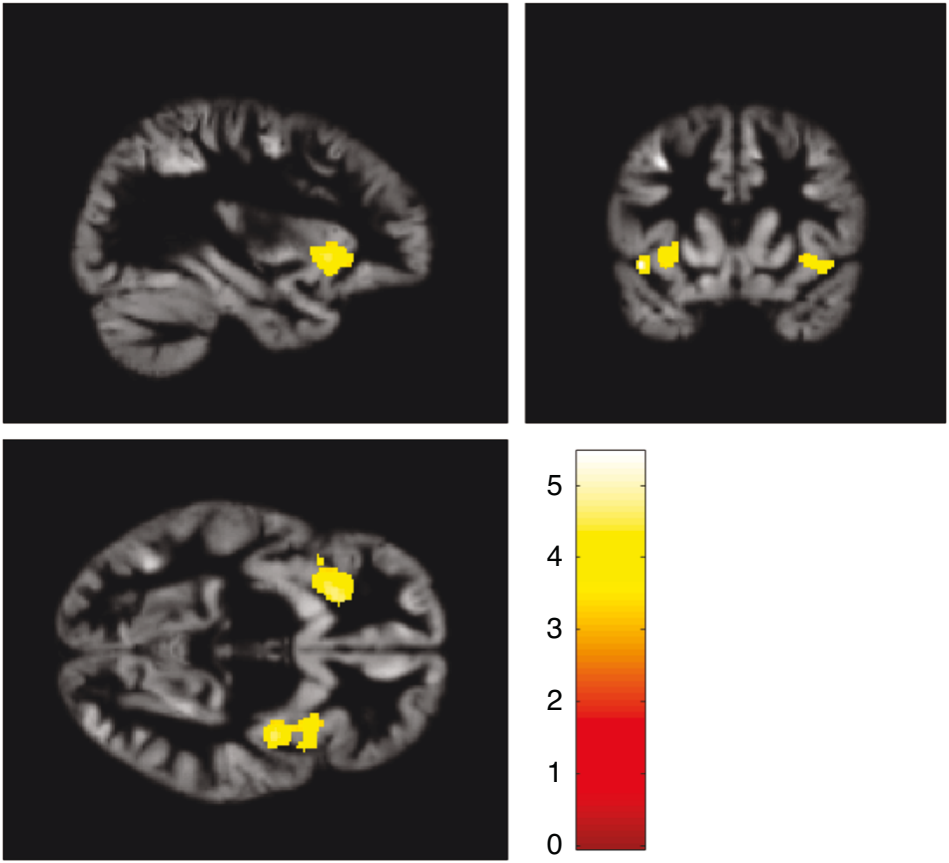
Regions in the bilateral insula where grey matter volume at 10 years was positively related with full-scale intelligence quotient for children born EPT (*n* = 42). Results are presented with a voxel-level threshold of *p* < 0.001, with family-wise error correction at cluster level. The color bar represents the t-statistics.

The images at 10 years of the 42 children born EPT were analyzed with VBM. This showed that GM volume in the left and right insula were positively correlated with FSIQ, when they were adjusted for sex, age at scan and maternal education (Table 4 and Fig. 3). These associations remained significant after adding GA at birth as a covariate, Table 4. There were no significant negative correlations between FSIQ and GM volume.

**Table 4:** Associations between grey matter volume at 10 years and FSIQ at 12 years for 42 children born extremely preterm analyzed with voxel-based morphometry.

Anatomical region	hemisphere	Cluster No. voxels	P-value	T statistics	Coordinates in MNI space		
					x	y	z
**Grey matter**							
**FSIQ, positive correlation, adjusted for sex, age at scan and maternal education**							
Insula	Left	764	0.006	5.45	**–**40	11	**–**5
Insula	Right	1223	<0.001	5.11	42	5	**–**5
**FSIQ, positive correlation, adjusted for sex, age at scan, maternal education and gestational age at birth**							
Insula	Left	550	0.025	5.12	**–**40	11	**–**5
Insula	Right	454	0.050	4.51	38	10	1

Presented clusters are thresholded at *p* < 0.001 at voxel-level with family-wise error correction at cluster level. *MNI* Montreal Neurological Institute, *FSIQ* full-scale intelligence quotient.

We did not find any significant associations between regional WM volume and FSIQ in the EPT group.

There were no positive or negative associations between regional GM or WM volumes with FSIQ for the 29 term-born controls, when they were analyzed with VBM.

### Total brain volumes at term age and associations with cognitive outcomes at 12 years for children born EPT

The GM and WM brain volumes at term age and their associations with cognitive data for the 25 children born EPT with MRI data at term and cognitive data at 12 years of age, adjusted for sex, GA at MRI, and maternal education, are presented in Table 5. The brain volumes were not significantly associated with the cognitive outcomes. However, when also adjusted for GA at birth the associations between brain volumes at term and FSIQ were significant and in positive direction, but did not remain after correction for multiple comparisons, Supplementary Table 4c).

**Table 5:** Associations between grey matter and white matter volumes at term age with cognitive outcomes at 12 years for children born EPT.

Children born EPT *n* = 25						
	Grey matter			White matter		
	β	CI	*p-*value	β	CI	*p*-value
**FSIQ**	0.15	–0.023; 0.32	0.090	0.19	–0.020; 0.39	0.077
**Verbal comprehension**	0.14	–0.18; 0.46	0.39	0.15	–0.27; 0.56	0.49
**Visual spatial**	0.043	–0.22; 0.31	0.75	0.030	–0.33; 0.39	0.87
**Fluid reasoning**	0.17	–0.014; 0.36	0.070	0.22	–0.027; 0.46	0.082
**Working memory**	0.22	–0.059; 0.49	0.12	0.28	–0.11; 0.65	0.17
**Processing speed**	0.15	–0.12; 0.42	0.28	0.14	–0.22; 0.49	0.45

The β value represents change in the cognitive assessment score for every cm^3^ increase in brain volume. Generalized estimating equations, dependent variable: cognitive outcome, independent variable: brain tissue in cm^3^, adjusted for sex, gestational age at MRI and maternal education. *FSIQ* full-scale intelligence quotient, *EPT* extremely preterm.

### Brain volume growth and cognitive outcomes for the EPT subset with longitudinal MRIs

The growth of brain volumes for GM and WM volumes in the 20 children born EPT with longitudinal MRI data were not significantly associated with FSIQ or any of the WISC-V index scores at 12 years of age. The analyses were adjusted for sex, maternal education, and ages at the time of the MRI scans (Table 6), and the results remained after also controlling for GA at birth, Supplementary Table 4d).

**Table 6:** Associations between the growth of grey matter (Δ GM) and white matter (Δ WM) volumes from term age to 10 years with cognitive outcomes at 12 years for 20 children born EPT with longitudinal data.

Children born EPT n = 20						
	Δ Grey matter			Δ White matter		
	β	CI	*p-*value	β	CI	*p*-value
**FSIQ**	–0.028	–0.14; 0.083	0.62	–0.056	–0.19; 0.079	0.42
**Verbal comprehension**	–0.014	–0.14; 0.12	0.83	–0.085	–0.19; 0.020	0.11
**Visual spatial**	–0.086	–0.19; 0.016	0.10	–0.080	–0.22; 0.61	0.26
**Fluid reasoning**	–0.022	–0.13; 0.087	0.69	0.18	0.070; 0.11	0.69
**Working memory**	0.013	–0.10; 0.13	0.83	–0.026	–0.21; 0.16	0.78
**Processing speed**	–0.10	–0.21; 0.003	0.058	–0.11	–0.25; 0.028	0.12

The β value represents change in the cognitive assessment score for every cm^3^ increase in brain volume growth. Generalized estimating equations, dependent variable: cognitive outcome, independent variable: brain tissue growth in cm^3^, adjusted for sex, ages at MRI, and maternal education. *FSIQ* full-scale intelligence quotient, *EPT* extremely preterm.

## Discussion

This prospective study of a well-categorized population of children born EPT, examined alongside term-born controls, found that the volumes at 10 years of age were positively associated with cognitive outcomes at 12 years. There were negative associations between cortical thickness at 10 years of age and cognitive outcomes. Although the total brain volumes and cortical thickness at 10 years of age were reduced in the children born EPT, compared to the term-born controls, their associations with cognition followed similar patterns in the two groups.

The insular volume was associated with FSIQ in children born EPT, but not in term-born controls. Most analyses between term-age brain volumes and cognitive outcomes were non-significant. The volumetric growth from term age to 10 years of age for the 20 children with longitudinal MRI data was not associated with cognition at 12 years.

### Total brain volumes at 10 years and associations to cognition

The total GM and WM brain volumes at 10 years of age were positively associated with cognitive scores at 12 years in both children born EPT and term-born controls. Few previous studies have exclusively examined children born EPT, but positive associations between brain volumes in childhood and adolescence in those born very preterm, and cognitive outcomes, have been reported.^4,5,29^ We previously reported that children born EPT in this cohort had reduced brain volumes at 10 years of age, compared to term-born controls, and that WM was most affected.^6^ The present study did not find evidence of WM being any more associated with FSIQ than GM.

GM and WM are not isolated brain tissues and there are inseparable connections between the components of the neural circuits that are highly inter-dependent.^1^ Therefore it is not surprising that their associations with cognitive outcomes demonstrate similar patterns and directions.^14^ We are aware that WM volume does not reflect the microstructural properties of the brain, that could be further studied using diffusion-weighted imaging.^30^

We did not find evidence of any different associations between total brain volumes at 10 years and cognition in the two groups, because the interactions were insignificant. There are studies that have reported stronger associations between brain volumes and cognition for preterm-born children than for term-born children, and it has been hypothesized that the associations are stronger when insults generate deviations from the individual’s primed growth trajectory.^29,31,32^ Fig. 2 shows that the slope was steeper for the association between GM volume and FSIQ in children born EPT than for term-born controls. It might be that a larger sample size would have discerned differences in associations between the two groups.

Nevertheless, one of few studies on children born EPT also reported that the associations between brain tissue volumes and cognition were similar in the children born EPT and term-born children when they reached 18 years of age.^9^ The brains of children born EPT are not organized in the same way as term-born children, as they have different microstructural properties.^10,33–35^ That we did not find stronger associations in the EPT group may be due to the many underlying structural and organizational differences that have been associated with their impaired neurodevelopment.^33^

Large studies have established a modest, but robust, positive association between total brain volume and intelligence in healthy children and adults.^36,37^ However, it is important to consider that children can have similar cognitive abilities, even when they have a 50% difference in brain size. Thus, there are numerous other factors than brain size that contribute to the capacity and efficiency of the brain.^38^

### Cortical thickness at 10 years of age and associations with cognition

Our study found that cortical thickness in the right hemisphere was negatively associated with FSIQ and the visual-spatial index in both study groups, prior to correction for multiple comparisons. Only the associations with visual-spatial index remained after adjustments for GA at birth. These findings could reflect those children with developed pruning mechanisms experienced beneficial cognitive effect.

Previous studies of large populations have identified negative associations between cortical thickness and intelligence from 10 years of age in typically developing children.^16^ The negative associations we found in the children born EPT were also in line with previous studies of very preterm children.^17,39,40^ However, one study reported positive associations between cortical thickness and intelligence when very-low-birth-weight children reached early adulthood.^15^ It is important to take the developmental stage into account when interpreting associations between cortical thickness and cognition, because cortical thinning begins in early childhood.^14^ Disease states can also lead to both increased cortical thickness, due to insufficient pruning mechanisms, and decreased cortical thickness due to impaired early growth of neural populations in the cortical columns.^13^ In the study that reported positive associations between cortical thickness and outcomes the children were born as early as in the late 1980s, when less developed neonatal care could have led to more early insults and impaired initial growth of the cortex.^15^

The associations between cortical thickness and cognition and GM volume and cognition at 10 years of age were reversed. It has been demonstrated that the GM volume growth follows the trajectory of surface area, while cortical thickness has an earlier peak.^14^ It is plausible that the benefits of GM volume size are driven by surface area at this age, in line with previous studies.^16,32,40,41^

Only the right-sided associations between cortical thickness and cognitive outcomes were statistically significant in our study. There is functional right-sided hemispheric dominance in visual-spatial processing, which could explain why this association was stronger in the right hemisphere.^42^ In healthy adolescents, cortical thinning has been shown to be more prominent in the right fronto-temporal regions, thus a more efficient pruning in the right hemisphere could be more associated with cognition.^43^

### Regional brain volumes at 10 years and cognition studied with VBM

Insular GM volume positively was associated with FSIQ in children born EPT. The insula is part of the salience network and plays a role in high-level attention.^44^ Studies also reported that the volume of the insula was especially affected following preterm birth.^45,46^ However, none of these studies investigate children born EPT exclusively.^45,46^

### Term age brain volumes and cognition

We found no significant associations between term age brain volumes and cognition at 12 years in children born EPT when results were adjusted for sex, GA at MRI, and maternal education. When results were adjusted for birth GA the associations between GM, WM and FSIQ were significant, prior to correction for multiple comparisons. The sample size was only large enough to detect strong associations. Studies that relate brain volume data when children born preterm reach term age with cognitive data beyond early childhood are rare, and we are not aware of any studies that have associated brain volumes at term age in just children born EPT with cognitive outcomes as late as at 12 years. Studies with very preterm children have reported positive associations between brain volumes at term age and cognitive outcomes in early childhood.^3,4,47,48^ The insignificant associations for the cognitive domains could have been due to compensatory mechanisms during the long follow-up period.^31^

### Volumetric growth from term to 10 years and associations with cognition

There was no significant association between brain tissue growth from term age to 10 years of age and cognitive outcome at 12 years in the EPT group. The children born EPT with longitudinal MRI data were more mature and had fewer perinatal risk factors than the non-participants. This could have contributed to this finding. However, this result was in line with a study that investigated growth for children born very preterm from term age to seven years of age.^4^ Our longitudinal analysis subgroup was small and the impact of brain growth throughout childhood in those born EPT should be further investigated in a larger population. It is important to note that total brain volume growth analyses do not rule out possibly important regional growth patterns.^12^

### Strengths and limitation

A strength of the study was the long follow-up period from EPT birth to 12 years of age. The study provided a broad perspective on associations between structural brain measurements and cognitive outcomes by comparing children born EPT, without major brain lesions, with term-born controls.

The study also had several limitations. The sample size was relatively small. However, the trends and exploratory findings in this under-researched patient group of children born EPT are still of interest.

The limited sample size also precluded using multivariable models that could be adjusted for more potentially relevant covariates. There were multiple possible confounding factors that could have influenced brain growth and cognition over so many years. But, we did adjust the data for the covariates that have been used most frequently in similar MRI studies.^49^

## Conclusions

The findings in this study indicate that brain volumes and cortical thickness assessed at 10 years are related to cognition at 12 years in children born EPT. Children born EPT had reduced brain volumes and cortical thickness at 10 years of age when they were compared with term-born controls, but their associations with cognitive outcomes followed similar patterns in the two groups. Most associations between term age brain volumes and cognitive outcomes were insignificant, and volumetric growth was not related with cognition for children born EPT. However, these associations need to be explored in larger studies of children born EPT. Insular volume may be relevant as a biomarker for cognitive outcomes in children born EPT.

## Supplementary information

Supplementary material

## Data Availability

Full datasets generated during and/or analyzed during the current study are available from the corresponding author on reasonable request.

## References

[1] Volpe, J. J. Dysmaturation of premature brain: Importance, cellular mechanisms, and potential interventions. *Pediatr. Neurol.* **95**, 42–66 (2019). 30975474 10.1016/j.pediatrneurol.2019.02.016

[2] Serenius, F. et al. Neurodevelopmental outcomes among extremely preterm infants 6.5 years after active perinatal care in Sweden. *JAMA Pediatr.* **170**, 954–963 (2016). 27479919 10.1001/jamapediatrics.2016.1210

[3] Keunen, K. et al. Brain tissue volumes in preterm infants: prematurity, perinatal risk factors and neurodevelopmental outcome: a systematic review. *J. Matern Fetal Neonatal Med* **25**, 89–100 (2012). 22348253 10.3109/14767058.2012.664343

[4] Monson, B. B. et al. Examination of the pattern of growth of cerebral tissue volumes from hospital discharge to early childhood in very preterm infants. *JAMA Pediatr.* **170**, 772–779 (2016). 27368090 10.1001/jamapediatrics.2016.0781

[5] de Kieviet, J. F., Zoetebier, L., van Elburg, R. M., Vermeulen, R. J. & Oosterlaan, J. Brain development of very preterm and very low-birthweight children in childhood and adolescence: a meta-analysis. *Dev. Med. Child Neurol.* **54**, 313–323 (2012). 22283622 10.1111/j.1469-8749.2011.04216.x

[6] Kvanta, H. et al. Exploring the distribution of grey and white matter brain volumes in extremely preterm children, using magnetic resonance imaging at term age and at 10 years of age. *PloS one* **16**, e0259717 (2021). 34739529 10.1371/journal.pone.0259717 PMC8570467

[7] Skiöld, B. et al. Sex differences in outcome and associations with neonatal brain morphology in extremely preterm children. *J. Pediatr.* **164**, 1012–1018 (2014). 24530122 10.1016/j.jpeds.2013.12.051

[8] Nosarti, C. et al. Grey and white matter distribution in very preterm adolescents mediates neurodevelopmental outcome. *Brain: J. Neurol.* **131**, 205–217 (2008). 10.1093/brain/awm282 18056158

[9] Cheong, J. L. et al. Contribution of brain size to IQ and educational underperformance in extremely preterm adolescents. *PloS one* **8**, e77475 (2013). 24130887 10.1371/journal.pone.0077475 PMC3793949

[10] Kvanta, H. et al. Extreme prematurity and perinatal risk factors related to extremely preterm birth are associated with complex patterns of regional brain volume alterations at 10 years of age: a voxel-based morphometry study. *Front Neurol.* **14**, 1148781 (2023). 37273719 10.3389/fneur.2023.1148781 PMC10235462

[11] Whitwell, J. L. Voxel-based morphometry: an automated technique for assessing structural changes in the brain. *J. Neurosci.* **29**, 9661–9664 (2009). 19657018 10.1523/JNEUROSCI.2160-09.2009 PMC6666603

[12] Thompson, D. K. et al. Tracking regional brain growth up to age 13 in children born term and very preterm. *Nat. Commun.* **11**, 696 (2020). 32019924 10.1038/s41467-020-14334-9 PMC7000691

[13] Kelly, C. E. et al. Cortical growth from infancy to adolescence in preterm and term-born children. *Brain: J Neurol.* (2023). 10.1093/brain/awad348 PMC10994536 37816305

[14] Bethlehem, R. A. I. et al. Brain charts for the human lifespan. *Nature* **604**, 525–533 (2022). 35388223 10.1038/s41586-022-04554-y PMC9021021

[15] Bjuland, K. J., Løhaugen, G. C., Martinussen, M. & Skranes, J. Cortical thickness and cognition in very-low-birth-weight late teenagers. *Early Hum. Dev.* **89**, 371–380 (2013). 23273486 10.1016/j.earlhumdev.2012.12.003

[16] Schnack, H. G. et al. Changes in thickness and surface area of the human cortex and their relationship with intelligence. *Cereb. Cortex* **25**, 1608–1617 (2015). 24408955 10.1093/cercor/bht357

[17] Córcoles-Parada, M. et al. Born too early and too small: higher order cognitive function and brain at risk at ages 8-16. *Front Psychol.* **10**, 1942 (2019). 31551853 10.3389/fpsyg.2019.01942 PMC6743534

[18] Skiöld, B. et al. White matter changes in extremely preterm infants, a population-based diffusion tensor imaging study. *Acta Paediatr.* **99**, 842–849 (2010). 20132144 10.1111/j.1651-2227.2009.01634.x

[19] Wikland, K. A., Luo, Z. C., Niklasson, A. & Karlberg, J. Swedish population-based longitudinal reference values from birth to 18 years of age for height, weight and head circumference. *Acta Paediatr.* **91**, 739–754 (2002). 12200898 10.1080/08035250213216

[20] Gregory, K. E., Deforge, C. E., Natale, K. M., Phillips, M. & Van Marter, L. J. Necrotizing Enterocolitis in the premature infant: neonatal nursing assessment, disease pathogenesis, and clinical presentation. *Adv. Neonatal Care* **11**, 155–164 (2011); quiz 165-156. 21730907 10.1097/ANC.0b013e31821baaf4 PMC3759524

[21] Padilla, N., Alexandrou, G., Blennow, M., Lagercrantz, H. & Aden, U. Brain growth gains and losses in extremely preterm infants at term. *Cereb. Cortex* **25**, 1897–1905 (2015). 24488941 10.1093/cercor/bht431

[22] Ashburner, J. A fast diffeomorphic image registration algorithm. *NeuroImage* **38**, 95–113 (2007). 17761438 10.1016/j.neuroimage.2007.07.007

[23] Fischl, B. & Dale, A. M. Measuring the thickness of the human cerebral cortex from magnetic resonance images. *Proc. Natl Acad. Sci. USA* **97**, 11050–11055 (2000). 10984517 10.1073/pnas.200033797 PMC27146

[24] Fischl, B. Freesurfer. *NeuroImage* **62**, 774–781 (2012). 22248573 10.1016/j.neuroimage.2012.01.021 PMC3685476

[25] Wechsler, D., Pearson Education, I. & Psychological, C. *Wechsler Intelligence Scale for Children*. *5th Ed*. (PsychCorp, 2014).

[26] McKinnon, K. et al. Association of preterm birth and socioeconomic status with neonatal brain structure. *JAMA Netw. Open* **6**, e2316067 (2023). 37256618 10.1001/jamanetworkopen.2023.16067 PMC10233421

[27] Benjamini, Y. & Hochberg, Y. Controlling the false discovery rate: a practical and powerful approach to multiple testing. *J. R. Stat. Soc. Ser. B (Methodol.)* **57**, 289–300 (1995).

[28] Dewey, D. et al. Very preterm children at risk for developmental coordination disorder have brain alterations in motor areas. *Acta Paediatr.* **108**, 1649–1660 (2019). 30891804 10.1111/apa.14786

[29] Northam, G. B., Liegeois, F., Chong, W. K., Wyatt, J. S. & Baldeweg, T. Total brain white matter is a major determinant of IQ in adolescents born preterm. *Ann. Neurol.* **69**, 702–711 (2011). 21391229 10.1002/ana.22263

[30] Kelly, C. et al. Brain tissue microstructural and free-water composition 13 years after very preterm birth. *NeuroImage* **254**, 119168 (2022). 35367651 10.1016/j.neuroimage.2022.119168

[31] Johnson, M. H., Jones, E. J. & Gliga, T. Brain adaptation and alternative developmental trajectories. *Dev. Psychopathol.* **27**, 425–442 (2015). 25997763 10.1017/S0954579415000073

[32] Bjuland, K. J., Rimol, L. M., Løhaugen, G. C. & Skranes, J. Brain volumes and cognitive function in very-low-birth-weight (VLBW) YOUNG ADults. *Eur. J. Paediatr. Neurol.* **18**, 578–590 (2014). 24775377 10.1016/j.ejpn.2014.04.004

[33] Padilla, N. et al. Breakdown of whole-brain dynamics in preterm-born children. *Cereb. Cortex* **30**, 1159–1170 (2020). 31504269 10.1093/cercor/bhz156 PMC7132942

[34] Kelly, C. E. et al. Long-term development of white matter fibre density and morphology up to 13 years after preterm birth: a fixel-based analysis. *NeuroImage* **220**, 117068 (2020). 32585342 10.1016/j.neuroimage.2020.117068

[35] Padilla, N. et al. Disrupted resting-sate brain network dynamics in children born extremely preterm. *Cereb. Cortex* **33**, 8101–8109 (2023). 37083266 10.1093/cercor/bhad101 PMC10321088

[36] Deary, I. J., Cox, S. R. & Hill, W. D. Genetic variation, brain, and intelligence differences. *Mol. Psychiatry* **27**, 335–353 (2022). 33531661 10.1038/s41380-021-01027-y PMC8960418

[37] Pietschnig, J., Penke, L., Wicherts, J. M., Zeiler, M. & Voracek, M. Meta-analysis of associations between human brain volume and intelligence differences: how strong are they and what do they mean? *Neurosci. Biobehav. Rev.* **57**, 411–432 (2015). 26449760 10.1016/j.neubiorev.2015.09.017

[38] Lenroot, R. K. & Giedd, J. N. Brain development in children and adolescents: insights from anatomical magnetic resonance imaging. *Neurosci. Biobehav Rev.* **30**, 718–729 (2006). 16887188 10.1016/j.neubiorev.2006.06.001

[39] Mürner-Lavanchy, I., Rummel, C., Steinlin, M. & Everts, R. Cortical morphometry and cognition in very preterm and term-born children at early school age. *Early Hum. Dev.* **116**, 53–63 (2018). 29179056 10.1016/j.earlhumdev.2017.11.003

[40] Sølsnes, A. E. et al. Cortical morphometry and IQ in VLBW children without cerebral palsy born in 2003–2007. *Neuroimage Clin.* **8**, 193–201 (2015). 26106543 10.1016/j.nicl.2015.04.004 PMC4473819

[41] Rimol, L. M. et al. Atypical brain structure mediates reduced IQ in young adults born preterm with very low birth weight. *NeuroImage* **266**, 119816 (2023). 36528311 10.1016/j.neuroimage.2022.119816

[42] Ocklenburg, S. & Güntürkün, O. Hemispheric asymmetries: the comparative view. *Front Psychol.* **3**, 5 (2012). 22303295 10.3389/fpsyg.2012.00005 PMC3266613

[43] Liao, Z. et al. Hemispheric asymmetry in cortical thinning reflects intrinsic organization of the neurotransmitter systems and homotopic functional connectivity. *Proc. Natl Acad. Sci. USA* **120**, e2306990120 (2023). 37831741 10.1073/pnas.2306990120 PMC10589642

[44] Uddin, L. Q., Nomi, J. S., Hébert-Seropian, B., Ghaziri, J. & Boucher, O. Structure and function of the human Insula. *J. Clin. Neurophysiol.* **34**, 300–306 (2017). 28644199 10.1097/WNP.0000000000000377 PMC6032992

[45] Ji, W. et al. Preterm birth associated alterations in brain structure, cognitive functioning and behavior in children from the ABCD dataset. *Psychol. Med.*, 1–10 (2023). 10.1017/S0033291723001757 37365781

[46] Ma, Q. et al. Lower gestational age is associated with lower cortical volume and cognitive and educational performance in adolescence. *BMC Med* **20**, 424 (2022). 36329481 10.1186/s12916-022-02627-3 PMC9635194

[47] Cheong, J. L. et al. Brain volumes at term-equivalent age are associated with 2-year neurodevelopment in moderate and late preterm children. *J. Pediatr.* **174**, 91–97.e91 (2016). 27174146 10.1016/j.jpeds.2016.04.002

[48] Romberg, J. et al. MRI-based brain volumes of preterm infants at term: a systematic review and meta-analysis. *Archives of disease in childhood Fetal and neonatal edition* (2022). 10.1136/archdischild-2021-322846 PMC9411894 35078779

[49] Hyatt, C. S. et al. The quandary of covarying: a brief review and empirical examination of covariate use in structural neuroimaging studies on psychological variables. *NeuroImage* **205**, 116225 (2020). 31568872 10.1016/j.neuroimage.2019.116225

