# Supplementary material for: Brain volumes and cortical thickness and associations with cognition in children born extremely preterm
Source: Pediatr Res. 2024 Aug 21;97(2):655–63. doi: 10.1038/s41390-024-03480-1 (PMC12014504; doi:10.1038/s41390-024-03480-1)
Supplement: Supplementary file 1 — Supplementary material [file 41390_2024_3480_MOESM1_ESM.pdf]

## Supplementary material

| | Included children born EPT with MRI at 10 years and cognitive data at 12 years (n=42) | Children born EPT who declined participation or had low-quality MRI data at 10 years (n=50) | *p*-value |
|---|---|---|---|
| Gestational age, median (range) weeks | 25.6 (23.6–26.6) | 25.4 (23.3–26.6) | [b]0.066 |
| Birth weight, mean (SD), g | 839 (151) | 807 (168) | [a]0.34 |
| Sex male, n (%) | 21 (50) | 28 (56) | [c]0.57 |
| Maternal education, dichotomized, university level, n (%) | 27 (64) | 13/19 (68) | [c]0.75 |
| Handedness, righthanded, n (%) | 34 (81) | 31/39 (79) | [c]0.58 |
| Multiple births, n (%) | 7 (17) | 9 (18) | [c]0.87 |
| Antenatal steroids, n (%) | 39 (93) | 47 (94) | [d]1.0 |
| Postnatal steroids, n (%) | 4 (10) | 11 (22) | [d]0.16 |
| Patent ductus arteriosus, n (%) | 29 (69) | 38 (76) | [c]0.46 |
| Patent ductus arteriosus ligation, n (%) | 14 (33) | 16 (32) | [c]0.89 |
| Patent ductus arteriosus medically treated, n (%) | 27 (64) | 37 (74) | [c]0.31 |
| Sepsis, n (%) | 30 (71) | 43 (86) | [c]0.085 |
| Intraventricular haemorrhage grade 1-2, n (%) | 15 (36) | 19 (38) | [c]0.82 |
| Small for gestational age <2SD, n | 4 (10) | 5 (10) | [d]1.0 |
| Necrotizing enterocolitis, any stage, n (%) | 7 (17) | 7 (14) | [c]0.72 |
| Any retinopathy of prematurity, n (%) | 34/42 (81) | 37/46 (80) | [c]0.95 |
| WM abnormalities, none, n (%) | 24/39 (52) | 19/47 (40) | [e]0.051 |
| mild, n (%) | 14/39 (42) | 25/47 (43) | |
| moderate, n | 1/39 (2) | 3/47 (7) | |
| Bronchopulmonary dysplasia, n (%) | 17/41 (41) | 25/49 (51) | [c]0.37 |

**Supplementary Table 1. Drop-out analyses for the included children with MRI measurements at 10 years of age and cognitive assessments at 12 years (n=42) and children that declined participation or had insufficient MRI quality to be included.**

[a]Student's t-test, [b]Mann-Whitney U test, [c]Pearson chi-square, [d]Fisher's exact test, [e]Linear-by-linear association. MRI= magnetic resonance imaging, WM = white matter.

| | **Included children born EPT with MRI at term age and cognitive data at 12 years (n=25)** | **Children born EPT who declined participation or had low-quality MRI data at term age (n=82)** | ***p*-value** |
|---|---|---|---|
| Gestational age, median (range) weeks | 25.6 (24.3–26.6) | 25.5 (23.3–26.8) | [b]0.16 |
| Birth weight, mean (SD), g | 838 (136) | 809 (171) | [a]0.22 |
| Sex male, n (%) | 12 (48) | 50 (61) | [c]0.25 |
| Maternal education, dichotomized, university level, n (%) | 16 (64) | 25/37 (67) | [c]0.77 |
| Handedness, righthanded, n (%) | 20/24 (83) | 47/59 (80) | [d]0.89 |
| Multiple births, n (%) | 5 (20) | 14 (15) | [c]0.78 |
| Antenatal steroids, n (%) | 24 (96) | 76 (96) | [d]1.0 |
| Postnatal steroids, n (%) | 1 (4) | 15 (15) | [d]0.11 |
| Patent ductus arteriosus, n (%) | 18 (72) | 59 (72) | [c]0.99 |
| Patent ductus arteriosus ligation, n (%) | 8 (32) | 24 (29) | [c]0.79 |
| Patent ductus arteriosus medically treated, n (%) | 18 (72) | 56 (68) | [c]0.73 |
| Sepsis, n (%) | 19 (76) | 63 (77) | [c]0.93 |
| Intraventricular haemorrhage grade 1-2, n (%) | 8 (32) | 31 (37) | [c]0.60 |
| Small for gestational age <2SD, n | 2 (8) | 10 (12) | [d]0.73 |
| Necrotizing enterocolitis, any stage, n (%) | 3 (12) | 12 (15) | [d]1.0 |
| Any retinopathy of prematurity, n (%) | 20 (80) | 65/78 (83) | [c]0.70 |
| WM abnormalities, none, n (%) | 15 (60) | 37/71 (52) | [e]0.61 |
| mild, n (%) | 9 (36) | 32/71 (45) | |
| moderate, n | 1 (4) | 2/71 (3) | |
| Bronchopulmonary dysplasia, n (%) | 9 (36) | 35/77 (45) | [c]0.41 |

**Supplementary Table 2. Drop-out analyses for the included children with MRI measurements at term age and cognitive assessments at 12 years (n=25) and children that declined participation or had insufficient MRI quality to be included.**

[a]Student's t-test, [b]Mann-Whitney U test, [c]Pearson chi-square, [d]Fisher's exact test, [e]Linear-by-linear association. MRI= magnetic resonance imaging, WM = white matter.

| | Included children born EPT with longitudinal MRI data and cognitive data at 12 years (n=20) | Children born EPT who declined participation or had low-quality MRI data (n=72) | *p*-value |
|---|---|---|---|
| Gestational age, median (range) weeks | 26.3 (24.3–26.6) | 25.4 (23.3–26.6) | [b]0.009* |
| Birth weight, mean (SD), g | 858 (141) | 811 (165) | [a]0.13 |
| Sex male, n (%) | 10 (50) | 39 (54) | [c]0.74 |
| Maternal education, dichotomized, university level, n (%) | 13 (65) | 27/41 (66) | [c]0.95 |
| Handedness, righthanded, n (%) | 17 (85) | 48/61 (78) | [d]0.82 |
| Multiple births, n (%) | 4 (20) | 12 (17) | [d]0.73 |
| Antenatal steroids, n (%) | 19 (95) | 67 (93) | [d]1.0 |
| Postnatal steroids, n (%) | 0 (0) | 15 (21) | - |
| Patent ductus arteriosus, n (%) | 13 (65) | 54 (75) | [c]0.37 |
| Patent ductus arteriosus ligation, n (%) | 3 (15) | 27 (38) | [d]0.065 |
| Patent ductus arteriosus medically treated, n (%) | 13 (65) | 51 (71) | [c]0.62 |
| Sepsis, n (%) | 14 (70) | 59 (82) | [c]0.24 |
| Intraventricular haemorrhage grade 1-2, n (%) | 6 (30) | 28 (39) | [c]0.47 |
| Small for gestational age <2SD, n | 2 (10) | 7 (10) | [d]1.0 |
| Necrotizing enterocolitis, stage II-III, n (%) | 2 (10) | 12 (17) | [c]0.73 |
| Any retinopathy of prematurity, n (%) | 15 (75) | 56/68 (82) | [c]0.46 |
| WM abnormalities, none, n (%) | 11 (55) | 32/66 (48) | [e]0.69 |
| mild, n (%) | 8 (40) | 31/66 (47) | |
| moderate, n | 1 (5) | 3/66 (4) | |
| Bronchopulmonary dysplasia, n (%) | 4 (20) | 38/70 (54) | [c]0.010* |

**Supplementary Table 3. Drop-out analyses for the included children born EPT with longitudinal MRI data and cognitive assessments at 12 years (n=20) and children that declined participation or had insufficient MRI quality to be included.**

[a]Student's t-test, [b]Mann-Whitney U test, [c]Pearson chi-square, [d]Fisher's exact test, [e]Linear-by-linear association. MRI= magnetic resonance imaging, WM = white matter. *= significant at <0.05.

| Children born EPT and term-born controls n=71 (EPT n=42 and term-born n=29) | | | | | | |
|---|---|---|---|---|---|---|
| | Grey matter | | | White matter | | |
| | β | CI | *p*-value | β | CI | *p*-value |
| **FSIQ** | 0.085 | 0.027; 0.13 | **0.004*** | 0.12 | 0.038; 0.21 | **0.005*** |
| **Verbal comprehension** | 0.059 | –0.023; 0.14 | 0.16 | 0.050 | –0.063; 0.16 | 0.39 |
| **Visual spatial** | 0.091 | 0.043; 0.14 | **<0.001*** | 0.11 | 0.038; 0.19 | **0.003*** |
| **Fluid reasoning** | 0.052 | 0.001; 0.10 | 0.045* | 0.083 | 0.014; 0.15 | **0.018*** |
| **Working memory** | 0.083 | 0.026; 0.14 | **0.002*** | 0.12 | 0.043; 0.20 | **0.003*** |
| **Processing speed** | 0.079 | 0.015; 0.14 | **0.016*** | 0.15 | 0.039; 0.25 | **0.007*** |

**Supplementary Table 4 a): Associations between grey matter and white matter volumes at 10 years with cognitive outcomes at 12 years for the combined group of 42 children born extremely preterm and 29 term-born controls.**

The β value represents change in the cognitive assessment score for every $cm^3$ increase in brain volume. Generalized estimating equations, dependent variable: cognitive outcome, independent variable: brain tissue in $cm^3$; adjusted for group (EPT/control), sex, age at scan, gestational age at birth and maternal education. *= significant at $p<0.05$, bolded values remained after correcting for multiple comparisons with the Benjamini-Hochberg procedure. FSIQ=full-scale intelligence quotient. EPT=extremely preterm.

| Children born EPT and term-born controls n=71 (EPT n=42 and term-born n=29) | | | | | | |
|---|---|---|---|---|---|---|
| | Mean cortical thickness, left | | | Mean cortical thickness, right | | |
| | β | CI | *p*-value | β | CI | *p*-value |
| **FSIQ** | –2.6 | –7.2; 2.0 | 0.27 | –4.0 | –8.3; 0.36 | 0.072 |
| **Verbal comprehension** | –2.0 | –7.9; 3.9 | 0.51 | –3.7 | –9.5; 2.0 | 0.20 |
| **Visual spatial** | –3.1 | –7.7; 1.5 | 0.19 | –4.6 | –9.0; –0.23 | 0.039* |
| **Fluid reasoning** | –1.2 | –4.4; 1.9 | 0.45 | –2.0 | –4.9; 0.88 | 0.17 |
| **Working memory** | –1.3 | –5.0; 2.5 | 0.51 | –1.4 | –4.8; 2.0 | 0.41 |
| **Processing speed** | –1.0 | –5.2; 3.2 | 0.63 | –1.9 | –6.1; 2.4 | 0.39 |

**Supplementary Table 4 b): Associations between left or right hemisphere cortical thickness at 10 years with cognitive outcomes for 42 children born EPT and 29 term-born controls.**

The β value represents change in the cognitive assessment score for every 0.1 mm increase in cortical thickness. Generalized estimating equations, dependent variable: cognitive outcome, independent variable: cortical thickness in 0.1 mm, adjusted for group (EPT/control), sex, age at scan, gestational age at birth and maternal education. *= significant at p<0.05, bolded values remained after correction for multiple comparisons. FSIQ=full-scale intelligence quotient. EPT=extremely preterm.

| **Children born EPT n=25** | | | | | | |
|---|---|---|---|---|---|---|
| | Grey matter | | | White matter | | |
| | β | CI | *p*-value | β | CI | *p*-value |
| **FSIQ** | 0.20 | 0.018; 0.38 | 0.031* | 0.25 | 0.028; 0.48 | 0.028* |
| **Verbal comprehension** | 0.18 | –0.18; 0.54 | 0.33 | 0.19 | –0.27; 0.66 | 0.42 |
| **Visual spatial** | 0.087 | –0.19; 0.36 | 0.53 | 0.085 | –0.28; 0.45 | 0.65 |
| **Fluid reasoning** | 0.16 | –0.058; 0.37 | 0.15 | 0.19 | –0.055; 0.43 | 0.13 |
| **Working memory** | 0.20 | –0.11; 0.50 | 0.20 | 0.23 | –0.18; 0.65 | 0.27 |
| **Processing speed** | 0.23 | –0.020; 0.48 | 0.071 | 0.24 | –0.11; 0.60 | 0.18 |

**Supplementary Table 4 c): Associations between grey matter and white matter volumes at term age with cognitive outcomes at 12 years for children born EPT.**

The β value represents change in the cognitive assessment score for every $cm^3$ increase in brain volume. Generalized estimating equations, dependent variable: cognitive outcome, independent variable: brain tissue in $cm^3$, adjusted for sex, gestational age at MRI, maternal education and gestational age at birth. FSIQ=full-scale intelligence quotient. EPT=extremely preterm. *= significant at p<0.05, bolded values remained after correction for multiple comparisons.

| Children born EPT n=20 | | | | | | |
|---|---|---|---|---|---|---|
| | Δ Grey matter | | | Δ White matter | | |
| | β | CI | *p*-value | β | CI | *p*-value |
| **FSIQ** | 0.013 | –0.089; 0.11 | 0.81 | 0.26 | –0.015; 0.54 | 0.064 |
| **Verbal comprehension** | 0.004 | –0.14; 0.15 | 0.95 | 0.14 | –0.45; 0.74 | 0.64 |
| **Visual spatial** | –0.061 | –0.17; 0.044 | 0.26 | 0.20 | –0.12; 0.52 | 0.22 |
| **Fluid reasoning** | –0.034 | –0.16; 0.088 | 0.58 | 0.17 | 0.012; 0.35 | 0.067 |
| **Working memory** | 0.009 | –0.14; 0.16 | 0.91 | 0.21 | –0.26; 0.67 | 0.38 |
| **Processing speed** | –0.050 | –0.17; 0.072 | 0.42 | 0.12 | –0.31; 0.55 | 0.59 |

**Supplementary Table 4 d): Associations between the growth of grey matter (Δ GM) and white matter (Δ WM) volumes from term age to 10 years with cognitive outcomes at 12 years for 20 children born EPT with longitudinal data.**

The β value represents change in the cognitive assessment score for every $cm^3$ increase in brain volume growth. Generalized estimating equations, dependent variable: cognitive outcome, independent variable: brain tissue growth in $cm^3$, adjusted for sex, ages at MRI, maternal education and gestational age at birth. FSIQ=full-scale intelligence quotient. EPT=extremely preterm.

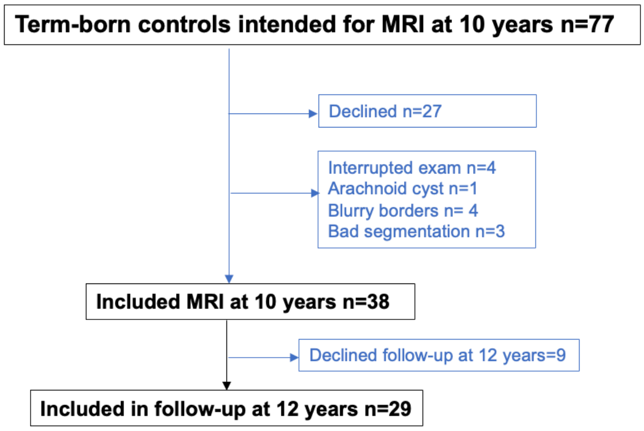


**Supplementary Figure 1. Flow chart of term-born controls with complete MRI data at 10 years of age and follow-up at 12 years of age.**

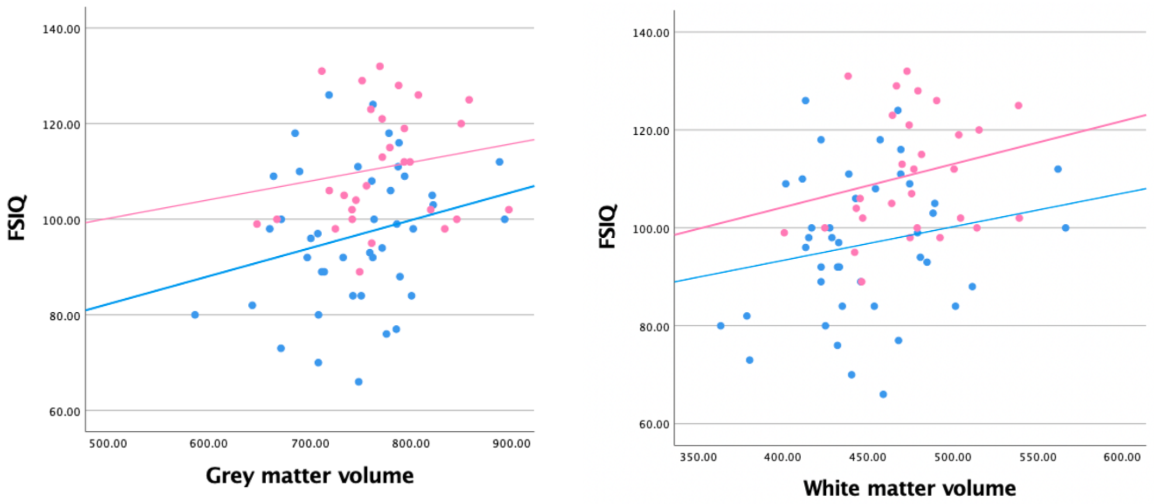


**Supplementary Figure 2. Scatterplots of raw grey matter and white matter volumes at 10 years and full scale IQ (FSIQ) at 12 years for 42 children born EPT (blue) and 29 term-born controls (pink).**
